# The Function of the Thyroid Gland

**Published:** 1885-10

**Authors:** 


### The Function of the Thyroid Gland.

Most works on physiology pass over the thyroid gland with a very superficial mention. It is said to exercise some part of importance in fœtal life—no one knows what. In extra-fœtal life, it is said to partially atrophy, and to be merely a useless organ to the adult — rather worse than useless, as in goitre it becomes inconvenient and sometimes dangerous.

This shows how little we know about human physiology. Recent researches have shown that the thyroid gland has an intimate and all-important relation to the highest functions of man—those of his brain. The fact was first developed by the extirpation of the gland in goitre, a proceeding which, according to the received views, ought to be wholly indifferent to the economy. Such is far from the case. After the total extirpation of the gland, the subjects steadily lose their mental vigor; the features become heavy; the speech slow and dull; the muscular system weakens, and the skin turns rough, thick and hard; in short, a condition gradually supervenes strikingly like that called by Charcot *myxœdema*, or the pachydermatic cachexia. They become *cretins.*

If ever so little of the gland remains, it is sufficient to prevent these changes; but its complete removal surely entails them. Experiments on dogs and cats yield similar results. The animals do not long survive, but are attacked with convulsions, somnolence and paralysis, which prove fatal.

Two theories have been advanced to explain these changes. One is that of Liebermeister, who maintains that the thyroid gland is the regulatory organ of the encephalic circulation, and that its abstraction throws this into chronic disorder. The other is that of Prof. Bruns, of Tübingen. He believes that the thyroid is either a depuratory gland which excretes certain substances poisonous to the nervous system, or that it fabricates certain substances indispensable to nervous vigor — which of the two, he is uncertain.

The very important practical conclusion remains uncontested, that, in all operations for goitre, a small portion of the gland should be allowed to remain.—*Phil. Med. and Surg. Reporter.*

